# Ameliorative effects of the traditional Chinese medicine formula *Qing-Mai-Yin* on arteriosclerosis obliterans in a rabbit model

**DOI:** 10.1080/13880209.2020.1803368

**Published:** 2020-10-18

**Authors:** Lei Zhang, Jia-Qin Yuan, Fu-Chen Song, Mei-Dong Zhu, Qi Li, Sheng-Hua Liu, Kai Zhao, Cheng Zhao

**Affiliations:** a Department of Vascular Surgery, Yueyang Hospital of Integrated Traditional Chinese and Western Medicine, Shanghai University of Traditional Chinese Medicine, Shanghai, PR China; b Yueyang Clinical Medical College, Shanghai University of Traditional Chinese Medicine, Shanghai, PR China; c Department of Traditional of Chinese Medicine, General Hospital of Ningxia Medical University, Yinchuan, PR China; d Department of Vascular Disease, Shanghai TCM-Integrated Hospital, Shanghai University of Traditional Chinese Medicine, Shanghai, PR China

**Keywords:** Inflammatory reactions, NF-κB signal, HPLC-Q-TOF-MS/MS, mechanisms

## Abstract

**Context:**

*Qing-Mai-Yin* (QMY) is a clinically used herbal formula for treating arteriosclerosis obliterans (ASO).

**Objective:**

To evaluate the chemical constituents and effects of QMY on ASO rabbit model.

**Materials and methods:**

Forty-eight New Zealand rabbits were divided into six groups (*n* = 8): normal (normal rabbits treated with 0.5% CMC-Na), vehicle (ASO rabbits treated with 0.5% CMC-Na), positive (simvastatin, 1.53 mg/kg), and QMY treatment (300, 600, and 1200 mg/kg). ASO rabbit model was prepared by high fatty feeding, roundly shortening artery, and bovine serum albumin immune injury. QMY (300, 600 and 1200 mg/kg) was orally administered for 8 weeks. The effects and possible mechanisms of QMY on ASO rabbits were evaluated by pathological examination, biochemical assays, and immunohistochemical assays. The compositions of QMY were analysed using HPLC-Q-TOF-MS/MS analysis.

**Results:**

Compared to the vehicle rabbit, QMY treatment suppressed plaque formation and intima thickness in aorta, and decreased intima thickness, whereas increased lumen area of femoral artery. Additionally, QMY treatment decreased TC, TG and LDL, decreased CRP and ET, and increased NO and 6-K-PGF1α in serum. Furthermore, the potential mechanisms studied revealed that QMY treatment could suppress expression of TNF-α, IL-6, ICAM-1 and NF-κB in endothelial tissues, and increase IκB. In addition, HPLC analysis showed QMY had abundant anthraquinones, stilbenes, and flavonoids.

**Conclusion:**

QMY has ameliorative effects on ASO rabbit, and the potential mechanisms are correlated to reducing inflammation and down-regulating NF-κB. Our study provides a scientific basis for the future application and investigation of QMY.

## Introduction

Arteriosclerosis obliterans (ASO) is one of the specific manifestations of systemic atherosclerosis (AS) in arteries, including arterial intimal thickening and stenosis or occlusion of artery lumen, resulting in deficiency of blood supply of body, and even life-threatening (You et al. 2010; Akagi et al. 2018; Halliday and Bax 2018). Epidemiological surveys have reported that currently approximately 200,000,000 patients are suffering from ASO worldwide (Halliday and Bax 2018; Zhang R et al. 2019). So far, thanks to the development of sciences and technology, the diagnosis of this disease has been improved greatly; however, the effective remedies of ASO are still limited. Currently, the available treatments for this disease mainly include surgery, such as endarterectomy, vascular bypass grafting, endovascular therapy, etc.; however, surgeries are suitable for only one third of the ASO patients, and this disease also has a high recurrence rate after simple surgery (Paumier et al. 2010; You et al. 2010). Besides, there are also some drugs for treating ASO, including vasodilatation drugs, antiplatelet drugs, thrombolytic drugs, anticoagulant drugs, etc.; however, the current drugs for ASO need long-term administration, and long-term use of these synthetic drugs would result in various bothersome side-effects, such as headache, bleeding, and serious diseases of digestive tract, etc. (You et al. 2010; Ding 2018). Consequently, discovery of more effective alternative remedies with low toxicities is beneficial for better management or treatment of this disease.

Traditional Chinese medicines (TCMs) have been used for management of various diseases in China for thousands of years. As natural herbal medicines, TCMs are commonly safe for people to use for fighting against many of difficult miscellaneous diseases (Zhang Q et al. 2019; Du et al. 2020; Long et al. 2020). *Qing-Mai-Yin* (QMY) is a clinically used TCM formula for treating AS, ASO, and dyslipidemia, etc. (Wang et al. 2004), consists of the roots of *Polygonum cuspidatum* Sieb. et Zucc. (Polygonaceae), whole herb of *Siegesbeckia orientalis* L. (Compositae), tuber of *Amorphophallus sinensis* Belval (Araceae), roots of *Rheum officinale* L. (Polygonaceae), whole herb of *Sedum sarmentosum* Bunge (Crassulaceae), *Laminaria japonica* Aresch (Laminariaceae), *Ostrea gigas* Thunb. (Ostreidae), and Ramulus of *Euonymus alatus* (Thunb.) Sieb (Celastraceae). Based on cytotoxic test and electron microscope determination, previous research revealed that QMY has protective effect on TNF-α stimulated vascular endothelial cells (VECs) against cell death, cell apoptosis and cellular structure damage (Zhang et al. 2013; Qian and Zhao 2014). However, there is no systemic study on QMY regarding its chemical compositions and its curative activities on ASO animal model as well as the related potential mechanisms. Consequently, our present study investigates the effects of *Qing-Mai-Yin* (QMY) on an ASO rabbit model and its potential mechanisms.

## Materials and methods

### Reagents and materials

Bovine serum albumin (BSA) was acquired from the Shanghai Huamei Biotech. Co. (Shanghai, China); cholesterol, sodium deoxycholate, and paraformaldehyde were purchased from the Sinopharm Chemical Reagent Co. Ltd. (Shanghai, China); pentobarbital sodium was provided by Sigma-Aldrich Co. (St. Louis, MO, USA); commercial detection kits for C-reactive protein (CRP), endothelin (ET), 6-K-prostaglandin (PG) F1α and nitric oxide (NO) were purchased from BOSTER Biotech. Co. (Wuhan, China); primary monoclonal antibodies for tumour necrosis factor (TNF)-α, interleukin (IL)-6, intercellular cell adhesion molecule (ICAM)-1, nuclear factor kappa-B (NF-κB) and inhibitor of NF-κB (IκB) were purchased from the SantaCruz Biotech. Inc. (Santa Cruz, CA, USA); Envision second reactants system was purchased from the DAKO Co. (Beijing, China); haematoxylin and eosin (H&E) was obtained from Solarbio Co. (Beijing, China).

### Animals

Forty-eight male New Zealand rabbits (8–10 weeks age, 2.3 ± 0.4 kg) were supplied by the experimental animal centre of Shanghai University of Traditional Chinese Medicine (Shanghai, China). The animal experimental protocols were handled according to the international guidelines for animal experiments, and approved by the Animal Care and Use Committee of Shanghai University of Traditional Chinese Medicine (Approval number PZSHUTCM190213002). Animals were housed in a quiet environment at 20–22 °C temperature and 40–50% humidity under a 12 h light/dark cycle and had free access to standard pellet diet and tap water. All the experiments were carried out after one week’s acclimatisation of the animals.

### Preparation of lyophilised powder of QMY

All eight herbal medicines were purchased from the Shanghai *Tongjitang* Pharmacy Ltd. Co. (Shanghai, China) in September 2018, and were identified by Prof. Hua Nian (Yueyang Hospital of Intergrated Traditional Chinese and Western Medicine, Shanghai University of Traditional Chinese Medicine). The voucher specimens of the herbal drugs were deposited at the Herbarium of our laboratory [the roots of *Polygonum cuspidatum* (no.QMY-01#PC), whole herb of *Siegesbeckia orientalis* (no.QMY-02#SO), tuber of *Amorphophallus sinensis* (no.QMY-03#AS), roots of *Rheum officinale* (no.QMY-04#RO), whole herb of *Sedum sarmentosum* (no.QMY-05#SO), *Laminaria japonica* (no.QMY-06#ST), *Ostrea gigas* (no.QMY-07#CO), and Ramulus of *Euonymus alatus* (no.QMY-08#RE)].

The 8 herbal medicines, including roots of *P. cuspidatum* (15 g), whole herb of *S. orientalis* (15 g), tuber of *A. sinensis* (15 g), roots of *R. officinale* (9 g), whole herb of *S. sarmentosum* (15 g), Sea-tangle (15 g), Concha Ostreae (15 g), and Ramulus of *E. alatus* (15 g), were refluxed with 8 times distilled water (1:8, w/v) in a glass flask (2 L) with glass condenser tube for 1 h. Then, the extracts were filtrated with gauze, and the filtrates were concentrated *in vacuo* under 60 °C with a rotary evaporator, and subsequently freeze-dried (yield rate was approximately 10%). Finally, the QMY extractives were powdered and kept in a dryer for the further experiments.

### ASO rabbit preparation and experimental protocols

The ASO rabbits were prepared according to the method reported by Wu et al. (2004) with minor modifications. From the start of the experiment, all the rabbits were fed with 100 g high-fat diet/day for 12 weeks which contains cholesterol (2%), sodium deoxycholate (1%), lard oil (10%) and normal feedstuff (87%) (Zhang et al. 2001). In the third day of the experiment, rabbits were intravenously injected of 3% pentobarbital sodium (3 mL/kg, i.v.). Under anaesthetisation, the femoral artery was isolated, and then the lumen of femoral artery was decreased by 1/3 using ligation with surgical sutures. Furthermore, all the rabbits received BSA (250 mg/kg, i.v.) by intravenous injection in the seventh day. Besides, another eight rabbits were treated as the normal control and fed with normal feedstuff, and were subjected to the same surgery procedure without ligation and BSA.

After 4 weeks’ feeding, the ASO rabbits were divided randomly into five groups (*n* = 8): Vehicle (ASO rabbits treated with 0.5% CMC-Na), Positive (ASO rabbits treated with simvastatin, 1.53 mg/kg/day), QMY (ASO rabbits treated with QMY at the doses of 300, 600 or 1200 mg/kg/day). Another 8 normal rabbits were used as the normal control (normal rabbits treated with normal saline). The doses of QMY and simvastatin were based on the used clinical dosage of human. The 0.5% sodium carboxymethyl cellulose (CMC-Na) solution was used to prepare the suspension of QMY and simvastatin, and all the drugs (20 mL) were administrated orally for consecutive 8 weeks.

### Sample collection

After a total of 8 weeks’ treatment, the rabbits were received anaesthesia with 3% pentobarbital sodium (3 mL/kg, i.v.) and were subsequently sacrificed prior to blood collecting from the abdominal aorta. In addition, the samples of right femoral artery and ascending aorta were resected and subsequently fixed in 4% paraformaldehyde.

### H&E staining

After being fixed in 4% paraformaldehyde for over 2 days, the artery samples were embedded in paraffin, and subsequently received a series of standard operations of section preparation, and finally sectioned into 5 μm tissue sections. After staining with H&E, the pathological changes of femoral artery and ascending aorta were evaluated under a BH2 microscope (Olympus, Tokyo, Japan).

### Sudan III staining

After being fixed in 4% paraformaldehyde for over 2 days, the artery tissue sections were cut using the way of frozen section, and washed with distilled water to remove the paraformaldehyde. The sections were dehydrated using gradient alcohol (50% and 70%), and subsequently the sections were stained with Sudan III solution for 30 min. The pathological changes of femoral artery and ascending aorta were evaluated under a BH2 microscope (Olympus, Tokyo, Japan).

### Biochemical analysis of serum lipid levels

Blood samples were centrifuged at 1000 × *g* at 4 °C for 15 min, and the supernatants were collected and kept at −80 °C before biochemical analysis. The levels of HDL-C, TC and TG in the serum were determined by a BT5370 automatic biochemical analyser (Biosistems Co., Spain), and the levels of LDL-C were calculated using the formula: 

LDL-C = TC－HDL-C－TG/2.2.

### Determination of CRP, ET, 6-K- PGF1α and NO levels

Blood samples were centrifuged at 1000 *g* at 4 °C for 15 min, and the supernatants were collected and kept at −80 °C before biochemical analysis. Serum levels of CRP, ET and 6-K-PGF1α were determined by ELISA methods according to the instructions of commercial kits with a microplate reader (Bio-Rad, USA) under 450 nm. In addition, NO levels were determined by nitrate reduction met using the commercial detection kits according to the standard experimental protocols of the manufactures’ instructions with a microplate reader (Bio-Rad, USA) under 550 nm.

### Immunohistochemical assays

The artery sections were dewaxed using dimethylbenzene and subsequently dehydrated using gradient alcohol. After washed with phosphate-buffered saline (PBS) three times (5 min each time), the sections were immersed in citrate buffer and then microwaved until boiling for two times (each time lasts 10 min). Then, the sections were washed twice with PBS (5 min each time), followed by blocking with goat serum at room temperature for 20 min, the tissue sections were incubated with primary antibodies (dilution 1:100) of TNF-α, IL-6, ICAM-1, NF-κB and IκB at room temperature for 1 h. The sections were washed with PBS for 3 times (5 min each time), then incubated with Envision second reactants for 1 h at room temperature. After washed with PBS three times (5 min each time), the 3,3′-diaminobenzidine (DAB) reagents were added to visualise the positive cells, and finally the haematoxylin dyeing located the cell nucleus. Images were captured under a BH2 microscope (Olympus, Tokyo, Japan) and subsequently analysed by using the Image J software (version: 1.51, National Institutes of Health, MD, USA).

### Qualitative HPLC-Q-TOF-MS/MS analysis of the QMY

HPLC-Q-TOF-MS/MS was utilised to qualitative determine the constituents of QMY. Chromatographic separation was performed on an Agilent 1290 HPLC system applying an XBridge BEH C_18_ chromatographic column (100 × 2.1 mm, 2.5 μm, i.d.) at 40 °C using a gradient elution at a flow rate of 1.0 mL/min. The mobile phase was consisted of 0.1% formic acid-water (A) and acetonitrile (B). The gradient programme was set as: 0–5 min, 5–20% B; 5–10 min, 20–40% B; 10–17 min, 40–75% B; 17–19 min, 75–100% B; and the sample injection volume was set as 2 μL. All MS experiments were conducted on an Agilent 6538 UHD Accurate - Mass Q - TOF LC/MS equipped with electrospray ionisation (ESI) interface. The MS analysis was carried out in negative scan mode, and the mass scan range was *m/z* 100–1000.

### Statistical analysis

All statistical analyses were carried out using the SPSS software (version 17.0, IBM Inc., USA). Data are showed as the mean ± SD. Differences among multiple groups were analysed by one−way ANOVA, followed by Least Significant Difference (LSD) *post hoc* test. *p* < 0.05 was considered as statistically significant.

## Results

### QMY suppressed plaque formation and intima thickness in aorta of ASO rabbits

**Figure 1.**
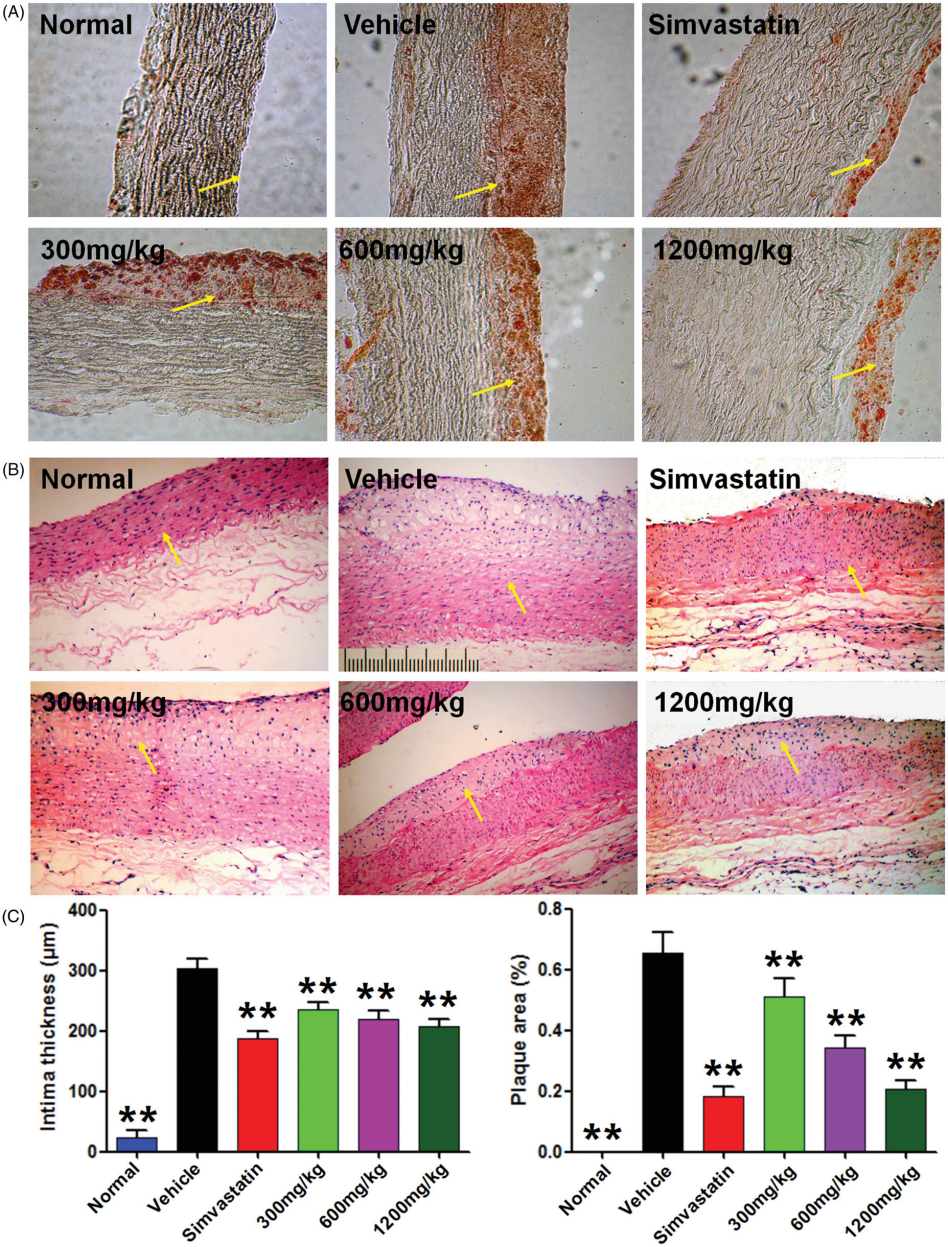
Effects of QMY on plaque formation and intima thickness in aorta of ASO rabbits. (A) Results of the Sudan III staining (magnification, ×100). (B) Results of the H&E staining (magnification, ×100). (C) Statistical results of the intima thickness and plaque area in aorta, data are represented as mean ± SD (*n* = 8), **p < 0.01 *vs*. vehicle. Simvastatin (1.53 mg/kg/day) was used as positive drug. Low, Middle and High doses of QMY were 300, 600, and 1200 mg/kg, and the simvastatin (1.53 mg/kg) was used as the positive drug. The yellow arrow shows the intima.

Sudan III is a good dyestuff for detection of aortic fatty embolism, and the fatty plaques in aorta could be stained in red (Xiong et al. 2013). As shown in Figure 1(A), aortic intima of normal rabbit is smooth and no obvious lesion (in red) could be observed; compared to the normal rabbit, large red area could be observed in aortic intima of ASO rabbit. However, treatment with positive drug and QMY could reduce the red area of aortic intima of ASO rabbit. In addition, similar results could be found in the H&E stained aortic tissue section showed in Figure 1(B,C), and the results revealed that in normal rabbit, intact and clear structures of the intima and vascular wall were observed. However, aortic intima of the ASO rabbit showed obvious lipid foam cell hyperplasia, exfoliation of endothelial cells, highly decreased aortic lumen, and serious increased intimal thickness, compared to the normal rabbit. Interestingly, treatment with QMY and positive drug can significantly ameliorate these pathological changes. Importantly, similar to the positive drug, QMY treatment (300, 600, 1200 mg/kg) can obviously suppress the plaque formation and intima thickness in aorta of ASO rabbits (*p* < 0.01), compared to the vehicle rabbit (ASO rabbit treated with normal saline).

### QMY suppressed intima thickness and increased the lumen area in femoral artery of ASO rabbits

**Figure 2.**
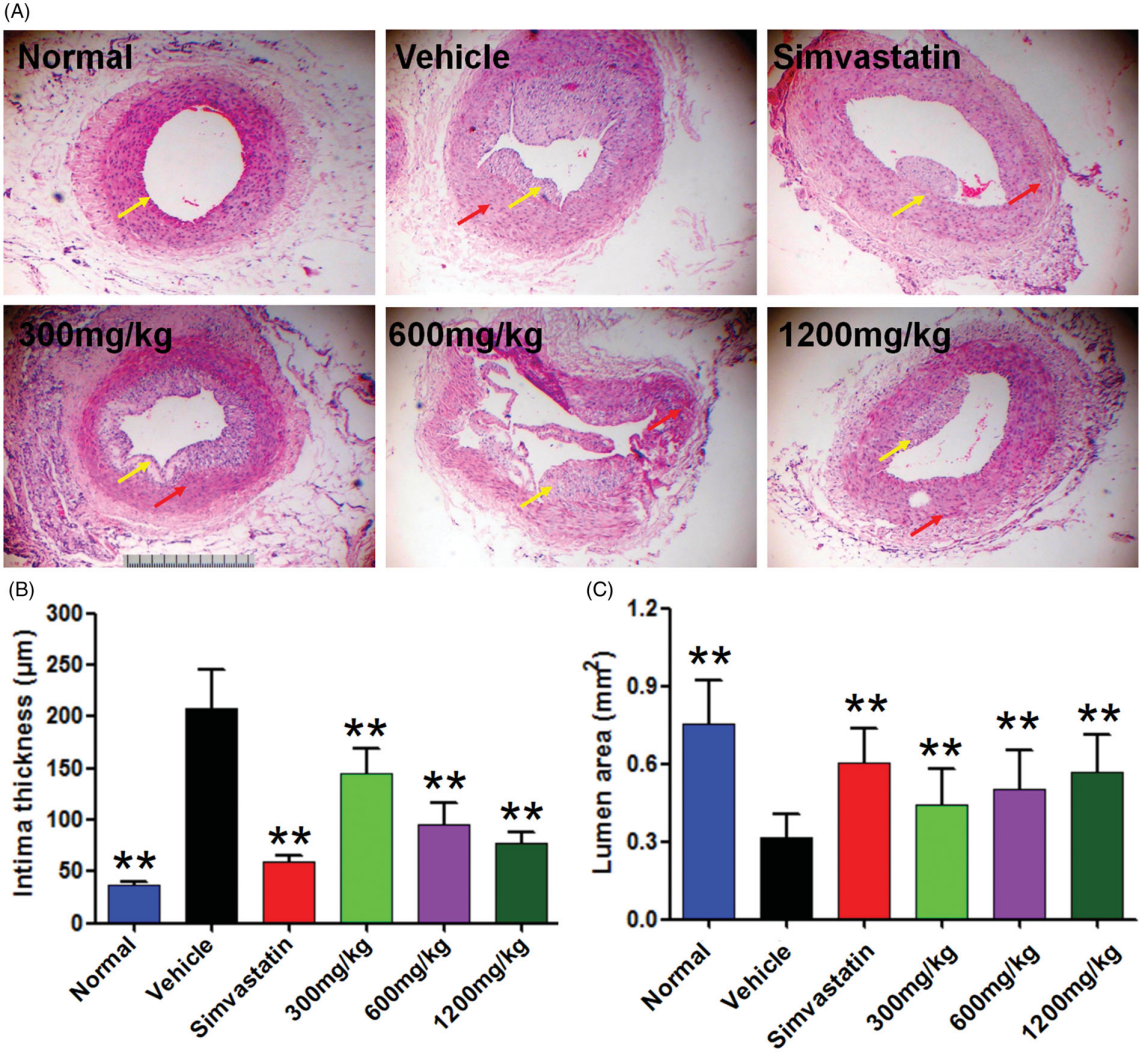
Effects of QMY on plaque formation and intima thickness in femoral artery of ASO rabbits. (A) Results of the HE staining (magnification, ×40). (B) Statistical results of the intima thickness and plaque area in aorta, data are represented as mean ± SD (*n* = 8), **p < 0.01 *vs*. vehicle. Simvastatin (1.53 mg/kg/day) was used as positive drug. Low, Middle and High doses of QMY were 300, 600, and 1200 mg/kg, and the simvastatin (1.53 mg/kg) was used as the positive drug. The yellow arrow shows the intima and the red arrow shows plaque.

In normal rabbit, the lumen of femoral artery is normal and no obvious stenosis could be seen, intima surface is smooth and no obvious plaque formed. Compared to the normal rabbit, intima of the femoral artery in ASO rabbit was markedly thickened (*p* < 0.01), the lumen area of the femoral artery was significantly decreased (*p* < 0.01), and obvious plaques could be observed which can be seen from the Figure 2. Interestingly, after treatment by positive drug and QMY, these pathological changes could be reversed. Results showed that QMY (300, 600 and 1200 mg/kg) could significantly decrease the intima thickness (*p* < 0.01) whereas increase the lumen area (*p* < 0.05, *p* < 0.01, *p* < 0.01, respectively) in femoral artery of ASO rabbits, compared to the vehicle rabbit (ASO rabbit treated with normal saline).

### QMY regulated the serum lipid levels of ASO rabbits

**Figure 3.**
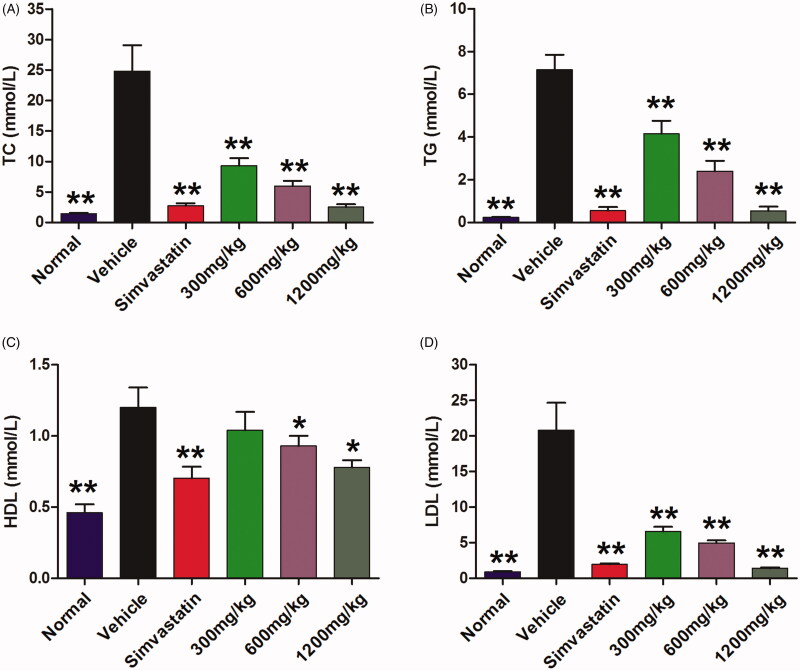
Effects of QMY on TC (A), TG (B), HDL (C), LDL (D) in serum of ASO rabbits. Data are represented as mean ± SD (*n* = 8), *p < 0.05, **p < 0.01 *vs*. vehicle. Simvastatin (1.53 mg/kg/day) was used as positive drug. Low, Middle and High doses of QMY were 300, 600, and 1200 mg/kg, and the simvastatin (1.53 mg/kg) was used as the positive drug.

Compared to the normal rabbit, the ASO rabbit showed higher serum levels TC, TG, HLD and LDL (*p* < 0.01). In contrary, the positive drug (*p* < 0.01) and QMY (300, 600 and 1200 mg/kg, *p* < 0.01) can significantly decrease the serum levels of TC, TG, and LDL, compared to the vehicle rabbit (ASO rabbit treated with normal saline). For the HDL, the QMY (600 and 1200 mg/kg, *p* < 0.05) also have slight reducing effects, compared to the vehicle rabbit (Figure 3).

### QMY decreased the CRP and ET and increased the NO and 6-K-PGF1αin serum of ASO rabbits

**Figure 4.**
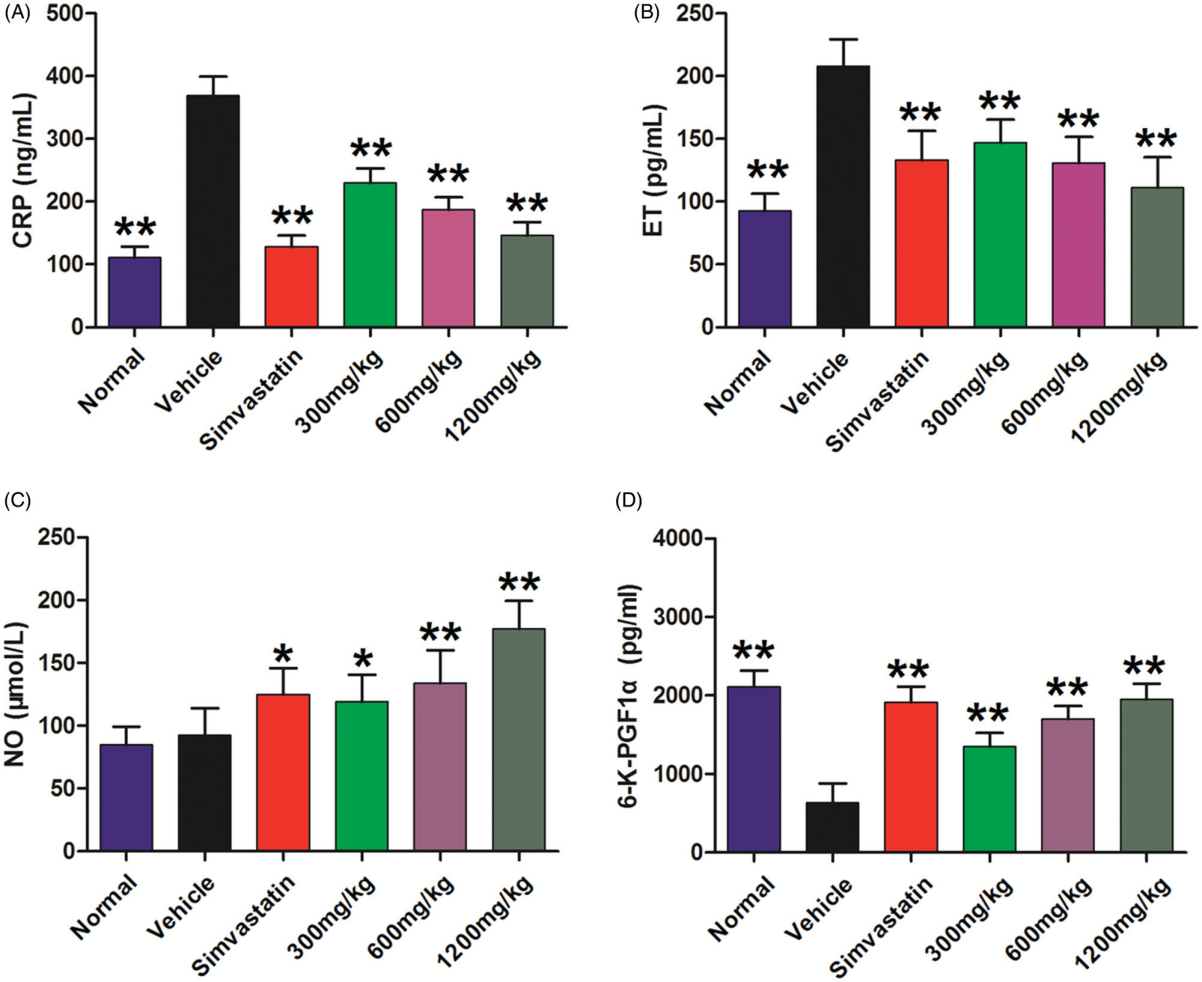
Effects of QMY on CRP (A), ET (B), NO (C), 6-K-PGF1α (D) in serum of ASO rabbits. Data are represented as mean ± SD (n = 8), *p < 0.05, **p < 0.01 *vs*. vehicle. Simvastatin (1.53 mg/kg/day) was used as positive drug. Low, Middle and High doses of QMY were 300, 600, and 1200 mg/kg, and the simvastatin (1.53 mg/kg) was used as the positive drug.

Furthermore, to investigate the potential mechanisms of QMY on ASO, we determined the effects of QMY on CRP, ET, NO, 6-K-PGF1α in serum of ASO rabbits. As shown in Figure 4, compared to the normal rabbit, the ASO rabbit showed remarkable higher levels of CRP (*p* < 0.01) and ET (*p* < 0.01) and lower levels of 6-K-PGF1α in serum. Furthermore, QMY at the doses of 300, 600 and 1200 mg/kg can significantly decrease the CRP (*p* < 0.01) and ET (*p* < 0.01), compared to the vehicle rabbit (ASO rabbit treated with normal saline). In contrary, similar to the positive drug, QMY (300, 600 and 1200 mg/kg) also increased the levels of 6-K-PGF1α (*p* < 0.01) and production of NO (*p* < 0.05, *p* < 0.01, *p* < 0.01) in serum of ASO rabbit compared to the vehicle rabbit.

### QMY decreased the protein expression of TNF-α, IL-6 and ICAM-1 in endothelial tissues of ascending aorta

**Figure 5.**
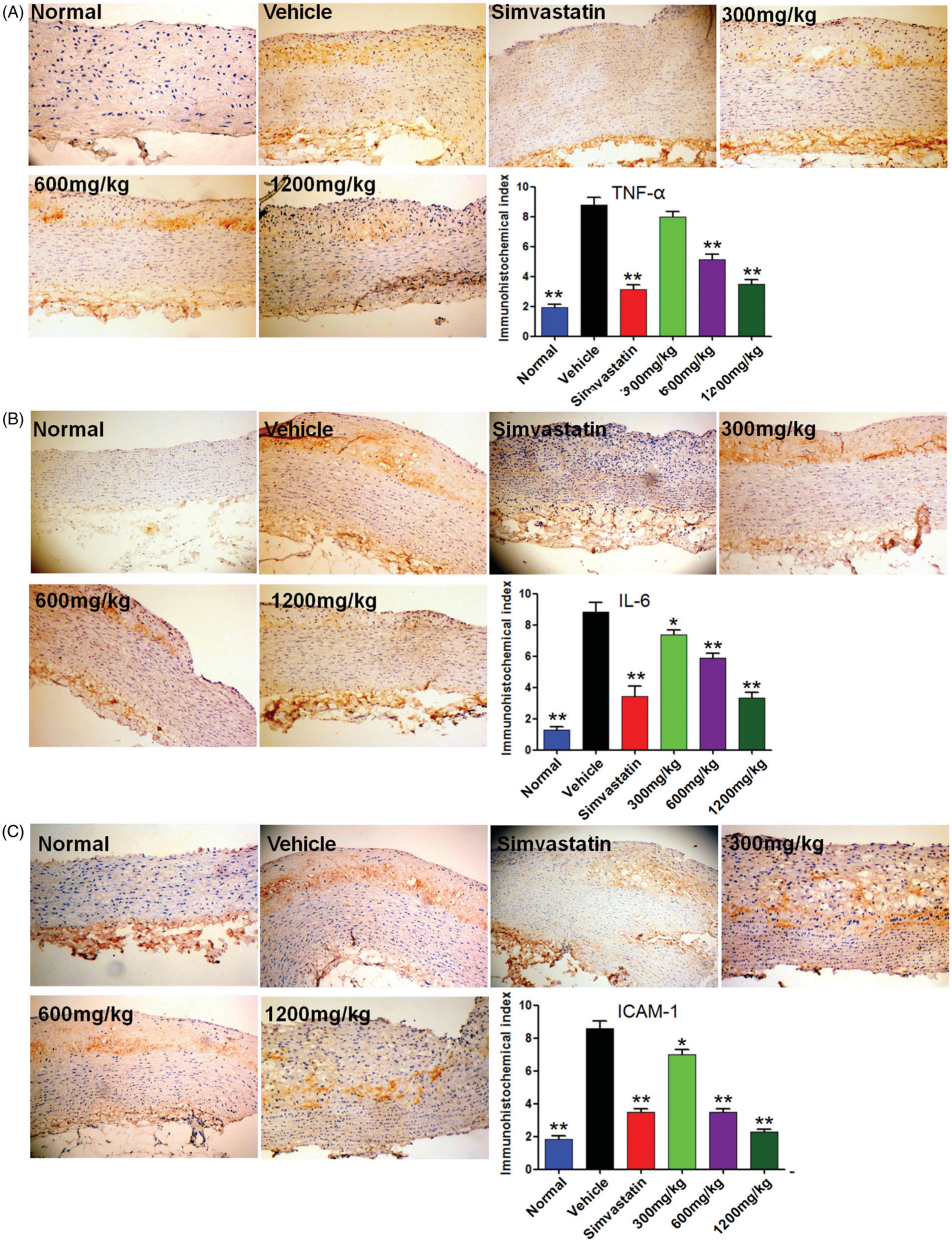
Effects of QMY on protein expression of TNF-α, IL-6 and ICAM-1 in endothelial tissues of ascending aorta (magnification, ×100). Data are represented as mean ± SD (*n* = 4), *p < 0.05, **p < 0.01 *vs*. vehicle. Simvastatin (1.53 mg/kg/day) was used as positive drug. Low, Middle and High doses of QMY were 300, 600, and 1200 mg/kg, and the simvastatin (1.53 mg/kg) was used as the positive drug.

We further investigated the production of inflammatory cytokines of TNF-α, IL-6 and ICAM-1 in endothelial tissues of ascending aorta. Compared to the normal rabbit, all these three inflammatory cytokines are highly over-expressed in endothelial tissues of ascending aorta of ASO rabbit (*p* < 0.01, Figure 5). Naturally, after treatment with the positive drug, all these inflammatory cytokines are down-regulated obviously (*p* < 0.01), compared to the vehicle rabbit (ASO rabbit treated with normal saline). Interestingly, our results also revealed that, similar to the positive drug, QMY (300, 600 and 1200 mg/kg) could decrease the expression of TNF-α (*p* < 0.01), IL-6 (*p* < 0.01) and ICAM-1 (*p* < 0.01) in endothelial tissues of ascending aorta of ASO rabbit, compared to the vehicle rabbit.

### QMY decreased the protein expression of NF-κB and increased IκB in endothelial tissues of ascending aorta

**Figure 6.**
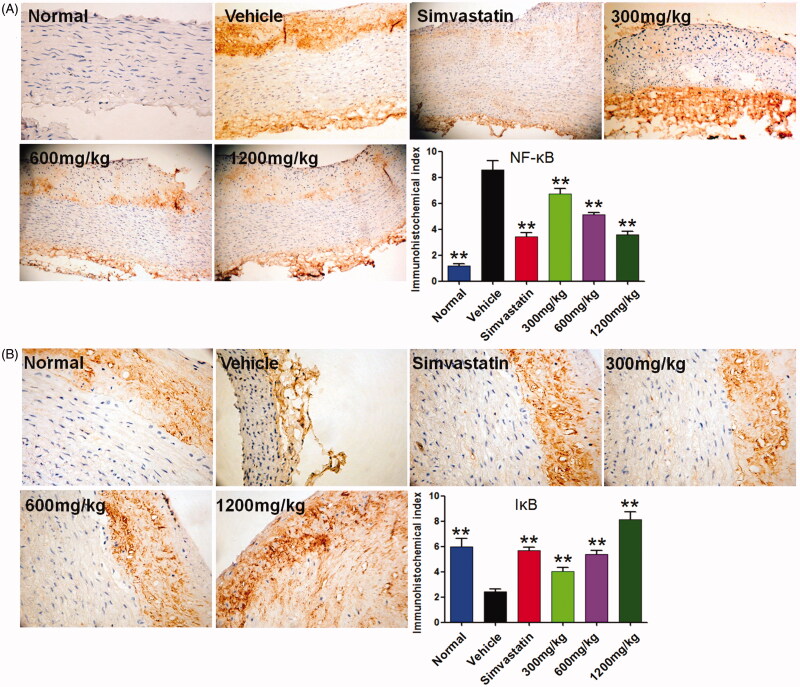
Effects of QMY on protein expression of NF-κB and IκB in endothelial tissues of ascending aorta (magnification, ×100). Data are represented as mean ± SD (*n* = 4), **p < 0.01 *vs*. vehicle. Simvastatin (1.53 mg/kg/day) was used as positive drug. Low, Middle and High doses of QMY were 300, 600, and 1200 mg/kg, and the simvastatin (1.53 mg/kg) was used as the positive drug.

Besides the inflammatory cytokines, we also studied the expression of NF-κB and IκB in endothelial tissues of ascending aorta. As shown in Figure 6, the ASO rabbit showed higher protein expression of NF-κB and lower protein expression of IκB in endothelial tissues of ascending aorta (*p* < 0.01), compared to the normal rabbit. In contrary, treatment with positive and QMY (300, 600 and 1200 mg/kg) could down-regulate NF-κB (*p* < 0.01) while up-regulate IκB (*p* < 0.01), in endothelial tissues of ascending aorta, compared to the vehicle rabbit (ASO rabbit treated with normal saline).

### Results of the HPLC-Q-TOF-MS/MS analysis of QMY

**Figure 7.**
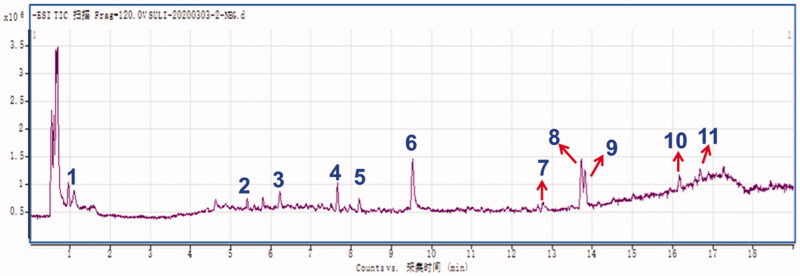
Result of the HPLC-Q-TOF-MS/MS assays of QMY. **1**–**11** represented the gallic acid (**1**), rutin (**2**), polydatin (**3**), resveratrol (**4**), kaempferol (**5**), emodin-8-*O*-glucoside (**6**), aloe-emodin (**7**), rhein (**8**), emodin (**9**), chrysophanol (**10**), and physcion (**11**), respectively.

Total ion chromatogram (TIC, negative mode) and the identified constituents of QMY were shown (Figure 7 and Table 1). A total of 11 compounds **1–11** with the retention time (RT) at 0.965, 5.418, 6.248, 7.686, 8.212, 9.535, 12.788, 12.734, 13.817, 16.183 and 16.709 min, were identified from the water extracts of QMY as gallic acid (**1**), rutin (**2**), polydatin (**3**), resveratrol (**4**), kaempferol (**5**), emodin-8-*O*-glucoside (**6**), aloe-emodin (**7**), rhein (**8**), emodin (**9**), chrysophanol (**10**), and physcion (**11**), respectively based on previous literature data (Xu et al. 2017; Zhang et al. 2017, 2018; Wang et al. 2018; Liu et al. 2019, 2020), and all 11 compounds were identified or deduced based on their precursor ions ([M-H]^-^) and characteristic MS/MS fragmental data. In addition, all the identified compounds were further confirmed by the available standard agents.

**Table 1.** Precursor and product ions of constituents in *Qing-Mai-Yin*.

No	*t*_R_ (min)	[M-H]^−^	MS/MS *m/z* (ESI^-^)	Identification
**1**	0.965	169.0147	125.0251, 79.0193, 51.0239	Gallic acid
**2**	5.418	609.1441	447.0949, 300.0285, 271.0251, 151.0039	Rutin
**3**	6.248	435.1292	227.0706, 185.0608,143.0512	Polydatin
**4**	7.686	227.0721	185.0613, 143.0492	Resveratrol
**5**	8.212	285.0408	256.0377, 229.1441, 171.0836, 139.1119, 112.9854,93.0361	Kaempferol
**6**	9.535	431.0983	269.0451, 225.0556, 197.0598	Emodin-8-O-glucoside
**7**	12.788	269.0448	239.0441, 223.0294, 195.0462, 183.0459, 67.0544	Aloe-emodin
**8**	13.734	283.0231	239.0352, 211.0407, 183.0449	Rhein
**9**	13.817	269.0452	241.0513, 225.0559, 197.0604	Emodin
**10**	16.183	253.0511	225.0573, 210.0334	Chrysophanol
**11**	16.709	283.0597	240.0431, 212.0506, 184. 0491	Physcion

## Discussion

To the best of our knowledge, this study is the first systemic animal evidence for demonstrating the ameliorative effects of *Qing-Mai-Yin* (QMY) on arteriosclerosis obliterans (ASO), and our present work also explored the related potential mechanisms of this TCM formula based on the animal model of ASO.

ASO is a common vascular disease, and its basic pathogenesis is arteriosclerosis (AS) (Zhang et al. 2002). Consequently, most of the ASO animal models are closely related to inducing arteriosclerosis. Based on a previous report on ASO animal models (Wu et al. 2004), we prepared the ASO rabbit model based on high fatty feeding, roundly shortening femoral artery and BSA immune injury. After successfully prepared the ASO rabbit, a large area of aortic fatty embolism, obvious lipid foam cell hyperplasia, exfoliation of endothelial cells, highly decreased aortic lumen, and serious increased intimal thickness can be observed in the artery. However, the results also revealed that QMY could reduce these mentioned pathological changes. QMY can suppress plaque formation and intima thickness in aorta, and inhibit intima thickness, as well as increase the lumen area in femoral artery of ASO rabbits. Lipid metabolism disorders especially hyperlipidaemia is recognised as the basic reasons for the development of AS, and increasing clinical evidences revealed that AS patients commonly have increased serum levels of TC, TG and LDL (Han et al. 2004; Wang et al. 2019). In our results, it’s interesting that QMY treatment could decrease the levels of TC, TG and LDL in serum of ASO rabbit. The over-produced LDL might be oxidised into oxidised LDL (ox-LDL), and the ox-LDL can further induce the releases of ICAM-1, pro-inflammatory cytokines (such as IL-6, TNF-α) and releases of ET, whereas decrease the releases of NO which is a protective factor for the endothelium cells (Lin et al. 2004; Liu and Zhang 2006; Han et al. 2007). In addition, the over-expressed inflammatory cytokines can further induce the production of CPR. All the inflammatory cytokines mentioned above including IL-6, TNF-α and CPR could directly injury and aggravates the injury of endothelial cells (Li and Li 2010; Zhao et al. 2010). ET is a strong vasoconstrictor and inflammatory media, whereas NO is the known active molecular for maintaining vasodilatation and protecting vascular endothelium (Davenport et al. 2016). The 6-K-PGF1α is derived from the PGI2 (a strong vasodilator), and can be used to reflect the PGI2 level (Yang et al. 2020). Endothelium injury plays crucial roles in the development of AS and ASO, and commonly presents high levels of ET, ICAM-1 and lower levels of NO and 6-K-PGF1α. In this study, we found that QMY treatment can decrease the levels of CRP and ET and increased the levels of NO and 6-K-PGF1α in serum of ASO rabbits. In addition, the present study also indicated QMY treatment is beneficial for suppression of protein expression of TNF-α, IL-6 and ICAM-1 in endothelial tissues of ascending aorta of ASO rabbits.

Previous research has suggested that inflammatory reactions are closely related to the development of AS, and suppression of inflammatory responses in the endothelial tissues would be beneficial for controlling the development of AS (Nasonov and Popkova 2018; Zhu et al. 2018). Our results suggested QMY exerted inhibitory effects on the releases of inflammatory cytokines and mediums. To explore the further potential molecular mechanisms of QMY, we investigated the proteins expression of NF-κB signal including NF-κB and IκB. In normal condition, NF-κB can be constrained by IκB, and stays in the cytoplasm in an inactivated form. However, the NF-κB could be activated by stimulations of inflammatory cytokines (such as TNF-α and IL-6) through disaggregation of the complex of NF-κB and IκB. Then, the activated NF-κB would transfer into the cell nucleus, resulting transcriptional reaction and expression of pro-inflammatory cytokines (Li et al. 2017). Interestingly, our results showed that QMY treatment could decrease the protein expression of NF-κB and increase IκB in endothelial tissues of ascending aorta, indicating suppression of NF-κB signal might be an important potential mechanism of QMY for its ameliorative effects on ASO.

Besides, we also determined the chemical compositions of this herbal formula. The HPLC-Q-TOF-MS/MS analysis showed that there are abundant anthraquinones, stilbenes and flavonoids in the QMY, including (1) anthraquinones: emodin, emodin-8-*O*-glucoside, aloe-emodin, rhein, emodin, chrysophanol and physcion; (2) stilbenes: polydatin and resveratrol; (3) flavonoids: rutin and kaempferol. All these mentioned natural agents have been reported from the herbal medicines of QMY, such as *P. cuspidatum*, *S. sarmentosum*, *R. officinale, S. orientalis*, etc. (Peng et al. 2013; Aichner and Ganzera 2015; Lin et al. 2015). As we know that anthraquinones, stilbenes and flavonoids are important natural agents for treating various diseases related to inflammatory responses, including AS and its related diseases (Fiorito et al. 2018; Li and Jiang 2018). Emodin and aloe-emodin have been reported to be beneficial for controlling hyperlipemia related diseases, and previous research also reported the two monomers possess good antioxidant and anti-inflammatory potential which are considered as one of the main molecular mechanisms of the two monmers for intervening various diseases (Wang et al. 2016; Bai et al. 2018; Devi et al. 2019; Xia et al. 2019). In addition, resveratrol is another known natural monomer in grapes and red wine with promising anti-aging and hypocholesterolemia activities, and the bioactivities of this compound are based on its significant antioxidant properties (Miura et al. 2003; Xia et al. 2017; Chassot et al. 2018). Interestingly, previous research have revealed that rutin possess good hypocholesterolemia and antioxidant effects, and can be found in various herbal medicines with hypocholesterolemia and antioxidant properties (da Silva et al. 2001; Li 2013; Merotto et al. 2017). However, more work in the future should be devoted to investigation of the potential active candidate agents from QMY against AS and ASO. In particular, it’s reported that flavonoids could affect the enzyme HMG-CoA reductase which could be beneficial for controlling the cholesterol synthesis in liver, and previous research also revealed that stilbenes and anthraquinones could reduce lipid accumulation via affecting the reabsorption of fats in the intestine (Niu et al. 2015; Ressaissi et al. 2017; Ye et al. 2019; Zhang YH et al. 2019). In our present study, we found that the QMY is rich in anthraquinones, flavonoids and stilbenes, suggesting that lipid metabolism regulation could be another important mechanism for the anti-ASO effect of QMY. Besides, in our previous study, we also found QMY could increase the SOD and CAT, whereas decrease the MDA (data not shown), thus antioxidant would be also a curial important mechanism for the anti-ASO effect of QMY.

## Conclusions

This study suggested that *Qin-Mai-Yin* (QMY) has potential ameliorative effects on arteriosclerosis obliterans (ASO) in a rabbit model, and the possible mechanisms of actions are related to reducing inflammatory responses and suppressing NF-κB signalling. Our study provides a scientific basis for the future application and investigation of QMY.
